# Management and Outcomes of Patients With Radiotherapy Interruption During the COVID-19 Pandemic

**DOI:** 10.3389/fonc.2021.754838

**Published:** 2021-11-19

**Authors:** Xiaofang Ying, Jianping Bi, Yi Ding, Xueyan Wei, Wei Wei, Fang Xin, Chuangying Xiao, Desheng Hu, Vivek Verma, Guang Han

**Affiliations:** ^1^ Department of Radiation Oncology, Hubei Cancer Hospital, Tongji Medical College, Huazhong University of Science and Technology, Wuhan, China; ^2^ Department of Radiation Oncology, The University of Texas MD Anderson Cancer Center, Houston, TX, United States

**Keywords:** COVID-19 pandemic, radiotherapy, interruption, outcomes, distant metastasis

## Abstract

**Purpose:**

This retrospective observational study examined patients who experienced radiotherapy (RT) interruption during the Wuhan lockdown for the novel coronavirus disease 2019 (COVID-19) pandemic.

**Materials and Methods:**

The data of all patients whose RT was interrupted during the Wuhan lockdown from January 23 to April 8, 2020 were collected. Patient-, cancer-, and treatment-related characteristics were analyzed, along with interruption time, disease progression type, and survival status. The methods employed in order to compensate for RT interruption were also described.

**Results:**

There were altogether 129 cancer patients whose RT was interrupted. Nineteen (14.7%) patients experienced a total interruption time of at most 7 days; the interruption time was 8–14 days for 27 (20.9%) patients, and 15 or more days for 47 (36.4%) patients. The remaining 36 (27.9%) patients did not come back to our hospital for further RT. We first describe our experience with re-immobilization and/or re-planning (n = 17) as well as dose compensation/adjustment. Of the 40 definitive radiotherapy patients, 37 had squamous cell carcinoma of nasopharyngeal, lung, or cervical origin. Most patients (85/93, 91.4%) were followed up for more than one year. Among the 40 patients who received definitive radiotherapy, nine patients experienced disease progression and five patients died. Three of the seven (42.9%) patients who did not finish radiotherapy after interruption died, as compared to only two of the 33 (6.1%) patients who completed radiotherapy. EQD2 (equivalent dose in 2 Gy fractions) at the time point of RT interruption was calculated. Five of the six patients (83.3%) who received EQD2 ≤10 Gy suffered from disease progression, compared with four of the 34 (11.8%) patients who received EQD2 >10 Gy. For the seven definitive radiotherapy cases who did not finish radiotherapy, three received systemic anti-cancer treatments and three died (all of whom did not receive further systemic therapies).

**Conclusions:**

This study provides the longest follow-up for the outcomes of RT interruption during COVID-19 pandemic to date. It cannot imply causation but implies that completing RT is important, along with the utility of having patients remain on systemic therapies if RT is to be interrupted.

## Introduction

The novel coronavirus disease 2019 (COVID-19) pandemic has affected virtually every aspect of health care, including the delivery of radiotherapy for cancer patients (1–4). Although cancer patients are more readily infected by COVID-19 due to weakened immune systems (5), delays or interruptions in cancer treatment can lead to tumor progression and/or poorer survival. Thus, balancing these risks is of utmost importance (6).

At the epicenter of the COVID-19 pandemic in Wuhan, China, many hospitals were utilized as COVID-19-designated hospitals, and hence many cancer patients’ treatment was interrupted or delayed. Our hospital is the only oncology-specific hospital in Wuhan, and thus we provided uninterrupted radiotherapy (RT) for cancer patients using an extensive departmental policy as described elsewhere (7). Although RT was not interrupted at our cancer center, cancer patients may not have been able to (or chose not to) come to our hospital during the Wuhan lockdown period (especially for patients coming from other cities in Hubei Province). Because delays/interruptions in RT are deleterious (8, 9), the COVID-19 pandemic has offered us a unique contemporary perspective to examine the outcomes of these patients, along with a description of the novel management approaches that were employed in this unique circumstance. This notion provided the impetus for this investigation, in which we examined patients at our cancer center who began RT before the Wuhan lockdown (January 23, 2020) and experienced various degrees of RT interruption.

## Materials and Methods

Data of all cancer patients who began RT at the Hubei Cancer Hospital before the Wuhan lockdown (from January 23, 2020 to April 8, 2020) and experienced any amount of treatment interruption were collected. Demographic, clinical and treatment characteristics were retrieved from the medical records and from our institution’s archiving systems. Data regarding the radiotherapy time, dose, fractionation, and planning systems was retrieved from the institutional Monaco and Eclipse systems. Patients were followed up for tumor progression and survival data (the last follow up date was May 9, 2021). EQD2 (equivalent dose in 2 Gy fractions) to the gross tumor volume at the time point of RT interruption was calculated. The overall treatment time (OTT) was calculated from the date of first day of radiotherapy to the day of radiotherapy completion. The follow up time was calculated from the date of radiotherapy completion to the date of last contact. PFS time was defined from the date that radiotherapy finished to the date of disease progression. Cross-tabulation and the Chi-squared test were used to compare categorical variables. All *p* values were two-sided, and *p <*0.05 was considered to be statistically significant. All statistical analyses were conducted with SPSS software package, version 19.0. This investigation was approved by the institutional ethics board of the Hubei Cancer Hospital of Huazhong University of Science and Technology in Wuhan, China.

## Results

**Figure 1:**
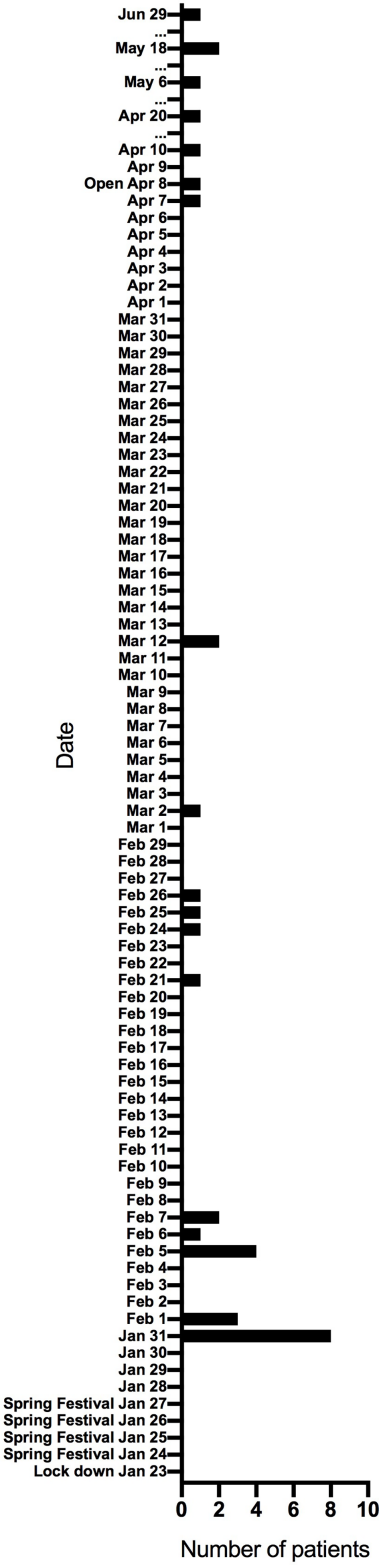
The date and number of patients who came back for further radiotherapy after radiotherapy was interrupted due to the COVID-19 pandemic.

There were 289 cancer patients who underwent radiotherapy in our hospital and 129 patients whose radiotherapy course was interrupted during the Wuhan lockdown. The most common reasons for RT interruption included fear of COVID-19 infection, travel inconvenience, tumor progression, and active/ongoing COVID-19 infection. We collected the date and number of patients who came back for further radiotherapy after initial interruption as shown in **Figure 1**, indicating that some patients experienced relatively long treatment breaks. **Table 1** displays characteristics of this population. The median interruption time was 15 days (range 7 to 157 days). Of note, the four most common tumors were cervical cancer (n = 27, 20.9%), breast cancer (n = 25, 19.4%), lung cancer (n = 20, 15.5%), and nasopharyngeal cancer (n = 16, 12.4%). A plurality of RT was delivered adjuvantly (n = 63, 48.8%), with the remainder as definitive (n = 40, 31.0%) or palliative (n = 26, 20.2%).

**Table 1:** Characteristics of patients who underwent interrupted radiotherapy.

Parameter, N (%)	Adjuvant radiotherapy (n = 63)	Definitive radiotherapy (n = 40)	Palliative radiotherapy (n = 26)
Sex			
Male	16 (25.4)	21 (52.5)	11 (42.3)
Female	47 (74.6)	19 (47.5)	15 (57.7)
Age (years)			
<65	55 (87.3)	27 (67.5)	18 (69.2)
≥65	8 (12.7)	13 (32.5)	8 (30.8)
Disease site			
Breast cancer	21 (33.3)	0 (0)	4 (15.4)
Lung cancer	0 (0)	8 (20.0)	12 (46.2)
Head and neck cancers	9 (14.3)	0 (0)	0 (0)
Nasopharyngeal cancer	0 (0)	14 (35.0)	2 (7.7)
Cervical cancer	12 (19.0)	14 (35.0)	1 (3.8)
Gastrointestinal cancers	7 (11.1)	0 (0)	1 (3.8)
All others	14 (22.2)	4 (10.0)	6 (23.1)
Interruption time (days)			
≤7	10 (15.9)	7 (17.5)	2 (7.7)
8-14	14 (22.2)	9 (22.5)	4 (15.4)
>14	23 (36.5)	17 (42.5)	7 (26.9)
Did not come back for further radiotherapy	16 (25.4)	7 (17.5)	13 (50.0)

Nineteen (14.7%) patients experienced a total interruption time of at most 7 days; the interruption time was 8–14 days for 27 (20.9%) patients, and 15 or more days for 47 (36.4%) patients. The remaining 36 (27.9%) patients did not come back to our hospital for further radiotherapy. Importantly, no patients who came back to continue radiotherapy became infected by COVID-19.

### Management of Patients Who Did Not Come Back For Radiotherapy

The detailed characteristics of patients who did not come back to our cancer center for further radiotherapy are summarized in **Table 2**. Of 36 patients, three patients went to another hospital for radiotherapy, 10 patients received systemic therapies (chemotherapy, targeted therapy, immunotherapy, or endocrine therapy), and 23 patients did not receive any further oncologic therapy. Of note, lung cancer was the most common tumor type for which other oncologic management was delivered (n = 6, 60%). Additionally, 22 patients (66.7%) from other cities in Hubei Province did not come back for radiotherapy, which was considerably higher than those living in Wuhan city (n = 11, 33.3%).

**Table 2:** The characteristics of patients who did not come back for radiotherapy.

	Radiotherapy at another hospital (n = 3)	Systemic therapy alone (n = 10)	No further cancer therapy (n = 23)
Sex, n (%)			
Male	1 (33.3)	4 (40.0)	13 (56.5)
Female	2 (66.7)	6 (60.0)	10 (43.5)
Age, n (%)			
<65 years	3 (100)	6 (60.0)	14 (60.9)
>=65 years	0 (0)	4 (40.0)	9 (39.1)
Tumor type, n (%)			
Breast cancer	0 (0)	1 (10.0)	1 (4.3)
Lung cancer	0 (0)	6 (60.0)	5 (21.8)
Head and neck cancer	0 (0)	0 (0)	5 (21.8)
Nasopharyngeal cancer	0 (0)	1 (10.0)	1 (4.3)
Cervical cancer	1 (33.3)	0 (0)	2 (8.7)
Gastrointestinal cancer	0 (0)	0 (0)	1 (4.3)
Others	2 (66.7)	2 (20.0)	8 (34.8)
Radiotherapy intent			
Definitive	0 (0)	3 (30.0)	4 (17.4)
Adjuvant	3 (100)	1 (10.0)	12 (52.2)
Palliative	0 (0)	6 (60.0)	7 (30.4)
Family address, n (%)			
Wuhan	2 (66.7)	4 (40.0)	7 (30.4)
Other cities in Hubei Province	1 (33.3)	6 (60.0)	16 (69.6)

Further follow up indicated that for 16 patients having started adjuvant radiotherapy, only one patient received other anti-cancer treatment (endocrine therapy for breast cancer; data not shown) and no patient died. For the seven definitive radiotherapy cases, three patients received other anti-cancer treatment (chemotherapy or immunotherapy) and three patients died (all of whom did not receive any further anti-cancer treatments). Of the 13 palliative cases, 6 patients received other anti-cancer treatment (chemotherapy, immunotherapy, or targeted therapy) and five patients died (all of whom did not receive other anti-cancer treatment).

### Replanning For Patients Who Came Back For Further Radiotherapy

There were 93 patients who came back for further radiotherapy after interruption. Replanning was done according to the discretion of the radiation oncologist, and most commonly was indicated due to anatomical- and/or tumor-related changes. A total of 17 (18%) patients received replanning: seven adjuvant cases, nine definitive cases, and one palliative case.

The immobilization apparatus had to be modified in several patients. Three head and neck patients wearing medical masks felt difficulty breathing from the initial thermoplastic mask, and as a result a new thermoplastic mask (with corresponding CT simulation and replanning) was made. One additional head and neck patient gained weight during radiotherapy interruption and the prior thermoplastic mask became too tight, resulting in resimulation and replanning. Three thoracic cancer patients’ immobilizations had to be changed to a vacuum-formed cradle and hence received new CT simulation and replanning. One breast cancer patient planned for breath-hold radiotherapy felt difficulty in holding her breath with a medical mask, and so simulation and replanning was done. Additionally, five patients whose radiotherapy interruption time was long (ranging from 88 to 130 days) received resimulation and replanning.

### Dose Compensation For Patients Who Come Back For Further Radiotherapy

**Figure 2:**
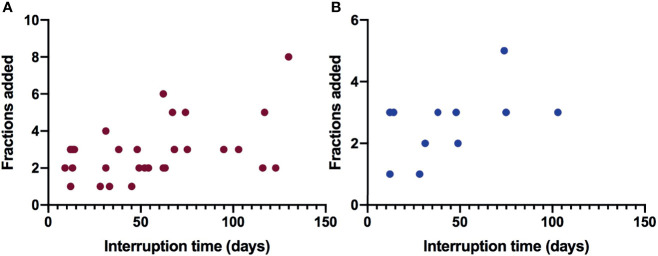
The relationship between interrupted time and dose compensation fractions for patients with interrupted radiotherapy. **(A)** Dose compensation for all cancer patients with interrupted radiotherapy. **(B)** Dose compensation for nasopharyngeal cancer patients with interrupted radiotherapy.

Among the 129 patients, most patients received 200 cGy/fraction radiotherapy except seven patients who were hypofractionated (300 cGy/fraction for bone metastasis, brain metastasis, or tumor thrombus of portal vein, and 800 cGy/fraction for lung cancer). No twice-daily or weekend radiotherapy was applied for compensation. Thirty-two patients received dose compensation to minimize the effect of radiotherapy interruption, among whom 12 also received replanning: 17 were adjuvant cases (seven breast cancer, seven head and neck cancer, three rectal cancer), 14 were definitive cases (12 nasopharyngeal cancer, two lung cancer) and one was palliative (lung cancer). The relationship between dose compensation and interruption time is shown by a scatter diagram for the whole patient (**Figure 2A**) and nasopharyngeal cancer patients (**Figure 2B**), respectively. Dose compensation was done according to the recommendations reported by Bese et al. (10), which suggested that the increase in dose required to maintain a constant local control rate is 0.5–0.6 Gy/d using 2 Gy fractions for head and neck cancer and 0.45 Gy/d for lung cancer. We also took other factors into account, including the interruption time, patients’ tolerance (including performance status), and the radiotherapy tolerance of the surrounding organs at risk. The final dose compensation was dependent on the radiation oncologist’s discretion as well as suggestions from the treatment team. Extra doses were administered once daily during working days, because twice-daily or weekend radiotherapy was difficult to apply during the COVID-19 pandemic. The largest dose compensation was for a rectal cancer patient undergoing adjuvant radiotherapy, for which eight fractions were added for an interruption time of 130 days. Adding five to six fractions were offered for four patients whose interruption time was more than 60 days. The most common compensation was adding two to three fractions for any degree of interrupted time. Twelve definitive nasopharyngeal cancer cases received dose compensation, and the number of additional fractions varied according to the radiation oncologist’s discretion as well as suggestions from the treatment team.

### Treatment Response of Definitive Radiotherapy With Interruption

Fourteen definitively-treated patients who returned for continuation of RT had imaging evaluation at the time they elected to re-start RT. According to the Response Evaluation Criteria for Solid Tumors (RECIST v1.1), 10 (71.4%) patients experienced a PR, three (21.4%) patients had SD, and only one (7.2%) patient suffered PD. Detailed information of the patients who received PR when they returned for continuation of RT is summarized in **Table 3**. There were two lung cancer (RT interruption time of 88 and 117 days, respectively) patients who had PR after only 16–24 Gy of EQD2 to GTV dose and two nasopharyngeal cancers (RT interruption time of 48 and 49 days, respectively) having received 15.2–25.2 Gy, indicating good radiosensitivity. Unfortunately, a nasopharyngeal cancer patient with cT3N2M0 (stage III, AJCC/UICC 8th) disease experienced PD. She received two cycles TP (docetaxel + cisplatin) induction chemotherapy and concurrent interrupted chemoradiotherapy. She had undergone only 11 Gy/5 fractions to the GTV before radiotherapy was interrupted for 103 days. MRI indicated a PR for the nasopharynx tumor but PD for a metastatic lymph neck node. The patient received subsequent concurrent chemoradiotherapy. Further follow up of that patient showed a PR for all areas of disease at one month after completion.

**Table 3:** RT details before radiotherapy interruption for patients who received PR when they returned for continuation of definitive RT.

	Tumor type	Stage	Pathological type	EQD2 to GTV(Gy)*	Radiation interrupted time (days)	Treatment before RT	Treatment concurrent with RT
Case1	Lung cancer	T4N3M0 IIIB	squamous cell	16.0	88	Chemotherapy	None
Case 2	Lung cancer	T3N2M0 IIIB	squamous cell	24.0	117	None	Chemotherapy
Case 3	Lung cancer	T4N2M0 IIIB	squamous cell	48.7	116	Chemotherapy	Chemotherapy
Case 4	Nasopharyngeal cancer	T2N1MO II	Non-keratinizing	25.2	49	Chemotherapy	Chemotherapy
Case 5	Nasopharyngeal cancer	T2N1M0 II	Non-keratinizing	55.9	74	None	Chemotherapy
Case 6	Nasopharyngeal cancer	T3N2M0 III	Non-keratinizing	15.2	48	Chemotherapy	Chemotherapy
Case 7	Nasopharyngeal cancer	T2N1M0 II	Non-keratinizing	46.4	38	Chemotherapy	Chemotherapy
Case 8	Nasopharyngeal cancer	T3N0M0 III	Non-keratinizing	50.7	31	Chemotherapy	Chemotherapy
Case 9	Cervical cancer	IIB	squamous cell	44.0	78	None	None
Case 10	Cervical cancer	IIIC1r	squamous cell	38.0**	157	Chemotherapy	Chemotherapy

*Following conversion of all dose-fractionation schemes to EQD2 based on the LQ model, assuming an α/β of 10. ** For cervical cancer, the radiotherapy dose for GTV refers to the external radiational dose, the patients also received internal radiotherapy at a dose of 12Gy/2 fractions for point A. EQD2, equivalent dose in 2 Gy fractions. GTV, gross tumor volume. PR, partial response. RT, radiotherapy.

### Outcomes of Patients With Interrupted Radiotherapy

As palliative radiotherapy patients often underwent other treatments such as chemotherapy, targeted therapy or immunotherapy, the effect of radiotherapy interruption on outcomes in that population was not analyzed. We therefore focused on the analysis of definitive and adjuvant radiotherapy.

The median follow up time (calculated from the date of radiotherapy completion to the date of last contact)was 14.2 months (range, 10.2–15.5 months). Most patients (85/93, 91.4%) were followed up for more than one year. The disease progression type, interrupted time, as well as overall treatment time is summarized in **Table 4**.

**Table 4:** The characteristics of definitive radiotherapy patients with disease progression or suffered death.

	Sex	Age (years)	Diagnosis	Stage	Histology	Dose (Gy)/ Fractions for the first course RT before interruption	Radiation interrupted time (days)	OTT (days)*	Relapse type	PFS (days) **
Case 1	male	54	Nasopharyngeal carcinoma	T3N2M0, III	Non-keratinizing	24/12	75	122	Liver metastasis	139
Case 2	female	52	Nasopharyngeal carcinoma	T3N2M0, III	Non-keratinizing	10/5	103	148	Liver metastasis	40
Case 3 #	male	67	Lung cancer	T4N2M0, III	Squamous cell	4/2	19	57	Regional tumor progression	141
Case 4 #&	male	67	Lung cancer	T4N2M0, III	Squamous cell	18/9	-	-	Regional tumor progression	63
Case 5 # &	male	70	Lung cancer	T4N0M0, III	Squamous cell	6/3	-	-	Regional tumor progression	325
Case 6	female	71	Cervical cancer	III	Squamous cell	10/5	32	67	Lung metastasis	8
Case 7#	female	72	Cervical cancer	IIIB	Squamous cell	16/8	8	44	None	-
Case 8 # &	female	81	Cervical cancer	IIIC2r	Squamous cell	10/5	-	-	Regional tumor progression	314
Case 9	female	61	Cervical cancer	IIIC2r	Squamous cell	20/10	7	48	Chest, abdominal and pelvic lymph node metastases	126

*OTT, overall treatment time. OTT was defined from the date of first day of radiotherapy started to the last day of radiotherapy finished; RT, radiotherapy; **PFS time was defined from the date radiotherapy finished to the date of disease progression. ^#^Patients died of cancer. ^&^Patients did not finish the radiotherapy after the interruption.

**Figure 3:**
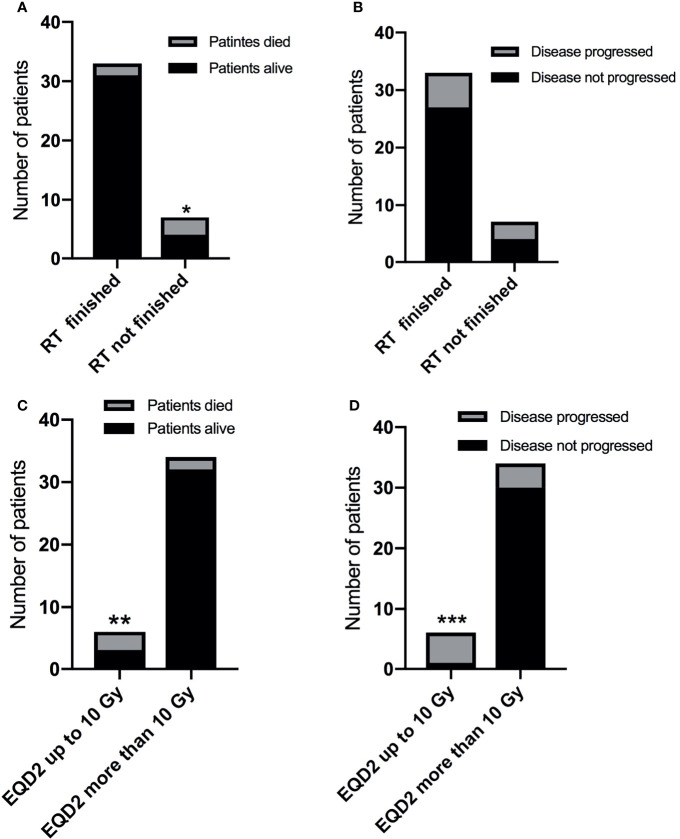
The impact of RT interruption on disease progression and survival. **(A)** The impact of radiotherapy completion on survival. *p = 0.008. **(B)** The impact of radiotherapy completion on disease progression. *P >*0.05. **(C)** The impact of gross tumor EQD2 (at the time point when RT was interrupted) on survival. **p < 0.001. **(D)** The impact of gross tumor EQD2 (at the time point when RT was interrupted) on disease progression. ***p = 0.003.

Among the 40 patients (37 of whom had squamous cell carcinoma of nasopharyngeal, lung, or cervical origin) who received definitive radiotherapy, nine patients suffered from disease progression and five patients died. Three of the seven (42.9%) patients who did not finish radiotherapy after interruption died, as compared to only two of the 33 (6.1%) patients who completed RT (**Figure 3A**, *p* = 0.008), while no difference was found between RT completion and disease progression (**Figure 3B**, *p* = 0.156).

EQD2 (at the time point of RT interruption) to the gross tumor volume was calculated. Five of the six patients (83.3%) who received an EQD2 ≤10 Gy prior to radiotherapy interruption suffered from disease progression, as compared with four of the 34 (11.8%) patients with an EQD2 >10 Gy (**Figure 3C**, *p <*0.001). The corresponding death rates were 3/6 (50%) patients versus 2/34 (11.8%) patients, respectively (**Figure 3D**, *p* = 0.003).

For the disease progression type, three patients who did not finish RT had regional disease progression, and four of the five patients who finished RT after interruption developed distant metastases. For the case #7 patient (cervical cancer, 72 years old) in **Table 4**, follow up data indicated that the patient died of severe thrombocytopenia which may have been due to pelvic radiotherapy and no tumor progression was detected. The patient went home after she finished RT on February 26, 2021. She was not able to monitor the blood count change due to inconvenient visiting to hospital during COVID-19 pandemic and suffered severe thrombocytopenia and died on April 30, 2021.

Among the 63 patients having received adjuvant radiotherapy, eight patients suffered from disease progression (four patients did not finish radiotherapy after interruption) but no patients died. One patient had local and regional (nodal) relapse, two with regional lymph node metastasis, and five developed distant metastasis.

## Discussion

The COVID-19 pandemic and the Wuhan lockdown provides a unique opportunity to evaluate radiotherapy interruptions in the contemporary era. There have been a few reports regarding this issue (11, 12), but management and outcomes of these patients remain scarcely reported in the literature to date.

We have previously reported that patient-perceived risks of infection and transportation-related difficulties were the main reasons for RT interruption during the COVID-19 pandemic (13). In this study, travel inconvenience was one of the most important factors that led to patients’ interruption of radiotherapy. Among the 36 patients who did not come back to our center for radiotherapy, 23 (63.9%) were from other cities in Hubei Province. There was a small proportion (n = 3, 8.3%) of patients who attempted to complete their radiotherapy at other hospitals, while other patients (n = 10, 27.8%) received other anti-cancer therapies (mostly systemic therapies). There were unfortunately relatively more (n = 23, 63.9%) patients who did not receive any further anti-cancer treatment, which may have affected local control and survival. Follow up indicated that for definitive and palliative radiotherapy, patients who neither came back for further radiotherapy nor received other oncologic treatment more often experienced disease progression and died. This indicates that for patients with active macroscopic tumor burden (i.e., definitive or palliative cases), it is still important to take measures to control tumor progression during radiotherapy interruption.

According to our center requirement, patients were required to wear a surgical mask for treatment delivery during the whole COVID-19 pandemic (14). It is reported that doing so is feasible and can provide basic protection for patients and staff during the COVID-19 pandemic (13). The impact of mask wearing on dosimetry is negligible (only 0.1 mm in water equivalent thickness) for patients requiring RT to the head and neck region, or clinical scenarios that require immobilization devices to that area, and has no impact even for proton beam therapy (15). However, anatomic and/or tumor-related disruptions from prolonged radiotherapy interruption still require compensation by means of replanning, which should be considered in each patient on an individualized basis.

Regarding compensation of the radiotherapy interruption, no twice-daily or weekend radiotherapy was applied, owing to its infeasibility during the COVID-19 pandemic. However, dose escalation was offered for 32 patients without changing the fractionation schemes. Fractionation scheme changes (mainly hypofractionated regimens) were more common for patients who needed to start their radiotherapy during COVID-19 pandemic (16). There are some suggestions regarding adjustment of radiation dose and fractionation to compensate for the short time of the radiotherapy interruption (17). However, there are no proper guidelines for patients with longer interruption times; as a result, these cases were managed according at the discretion of each radiation oncologist with input from the treatment team. A particularly interesting notion is whether patients whose tumors had a good response from the initial radiotherapy course (prior to interruption), presumably indicating favorable tumor biology, require dose escalation at all.

The preliminary follow up analysis for definitive radiotherapy (mainly squamous cell carcinoma of nasopharyngeal, lung, or cervical origin) indicated that patients who finished radiotherapy even after long interruptions have better prognoses than patients who did not continue their interrupted radiotherapy, especially for lung cancer patients. This is consistent with data from a report of curative-intent radiation therapy for anal cancer, wherein patients who did not complete chemoradiation had a higher risk of requiring salvage abdominoperineal resection, overall death, cancer-specific death, and colostomy or death (18). The follow up for patients with interrupted radiotherapy indicated that most common type of disease progression was distant metastases for both definitive and for adjuvant radiotherapy. This implies that chemotherapy or other systemic therapies may be particularly important during long radiotherapy interruptions. For patients who finished RT during the COVID-19 pandemic, RT complications should be a concern, which should be monitored carefully to avoid severe complication and death.

The limitation of this study is the heterogenous nature of the patient population, including the disease site, systemic treatments, and lack of a uniform standard for dose compensation. Additionally, our investigation was a retrospective observational study that is not optimally equipped to draw conclusions regarding the outcomes of patients with radiotherapy interruption.

## Conclusions

The COVID-19 pandemic and the Wuhan lockdown (January 23 to April 8, 2020) provides a unique opportunity to evaluate radiotherapy interruptions in the contemporary era. This study provides the longest follow up for the outcomes of RT interruption during COVID-19 pandemic to date. It cannot imply causation but nevertheless implies the importance to finish RT even after interruption as well as potentially having patients remain on systemic therapies if RT is to be interrupted. No patients who came back to continue radiotherapy became infected by COVID-19, indicating the safety to continue RT with proper protection measures. As the COVID-19 pandemic continues to impair adequate healthcare delivery, our experience may be utilized by other centers to manage the challenging clinical situation of interrupted radiotherapy.

## Data Availability Statement

The original contributions presented in the study are included in the article/supplementary material. Further inquiries can be directed to the corresponding authors.

## Ethics Statement

The studies involving human participants were reviewed and approved by the Ethics Committee of Hubei Cancer Hospital of Huazhong University of Science and Technology in Wuhan, China. Written informed consent for was obtained from the individual(s) for the publication of any potentially identifiable data included in this article, while for other participations, written informed consent was not required for this study in accordance with the national legislation and the institutional requirement.

## Author Contributions

All authors listed have made a substantial, direct, and intellectual contribution to the work and approved it for publication.

## Funding

This work was supported by the Hubei Provincial Health Commission (No. ZY2021M008).

## Conflict of Interest

The authors declare that the research was conducted in the absence of any commercial or financial relationships that could be construed as a potential conflict of interest.

## Publisher’s Note

All claims expressed in this article are solely those of the authors and do not necessarily represent those of their affiliated organizations, or those of the publisher, the editors and the reviewers. Any product that may be evaluated in this article, or claim that may be made by its manufacturer, is not guaranteed or endorsed by the publisher.
